# Optic Disc and Retinal Strain Due to Vertical Duction

**DOI:** 10.1167/iovs.67.11.15

**Published:** 2026-09-10

**Authors:** Atharva Shetye, Qingyu Meng, Emanuil Parunakian, Veronika Yehezkeli, Somaye Jafari, Joseph L. Demer

**Affiliations:** 1 Department of Ophthalmology and Stein Eye Institute, University of California, Los Angeles, Los Angeles, California, United States; 2 Mechanical and Aerospace Engineering Department, University of California, Los Angeles, Los Angeles, California, United States; 3 Department of Ophthalmology, Peking University People's Hospital, Beijing, China; 4 David Geffen School of Medicine, University of California, Los Angeles, Los Angeles, California, United States; 5 Department of Neurology, University of California, Los Angeles, Los Angeles, California, United States; 6 Neuroscience Interdepartmental Program, University of California, Los Angeles, United States; 7 Department of Bioengineering, University of California, Los Angeles, Los Angeles, California, United States

**Keywords:** eye movements, retinal vessels, deep learning, strain, optic disc, vertical duction

## Abstract

**Purpose:**

To compare two-dimensional optic disc and retinal vessel strains due to vertical versus horizontal eye rotations in younger and older subjects using confocal scanning laser ophthalmoscopy (cSLO).

**Methods:**

In a younger subject group (*n* = 10; range, 18–39 years; mean age ± SD, 30 ± 6 years) and an older subject group (*n* = 29; at least 40 years of age; mean age ± SD, 67 ± 10 years), high-resolution infrared cSLO images were acquired in central gaze, 10° supraduction and infraduction, and 30° abduction and adduction. Images were segmented using a deep-learning model to identify retinal vessels, optic disc, and fovea. Ocular torsion was corrected, and eccentric gaze images were registered to the central gaze image. Displacement fields between image pairs were estimated using Recurrent All-Pairs Field Transforms (RAFT) optical flow and converted to two-dimensional strains. Repeatability was assessed on nine additional subjects (five younger, three older, and one child 14 years old) by analyzing duplicate image acquisitions.

**Results:**

Equivalent strain within the optic disc reached ∼2% during all four eccentric gazes despite smaller vertical ductions and decreased rapidly with distance from the disc. Younger subjects exhibited approximately twice the vessel strain as older subjects for the same gaze angles. Horizontal strain reached about 1% during adduction and was greater in eyes with longer axial length. Shear strain reached about 0.48% during supraduction.

**Conclusions:**

Modest vertical ductions produce two-dimensional retinal vascular strains comparable to large horizontal ductions. Age and axial length significantly influence strain magnitude during duction, suggesting that resulting mechanical loading on the posterior eye might be more significant than previously appreciated.

The eye rotates thousands of times daily, both horizontally and vertically.^1^ These eye rotations range from tiny microsaccades that are necessary for optimal visual acuity^2^ to large voluntary gaze shifts mediated by the vestibulo-ocular reflex (VOR) during head movements.^3^ During eye rotations, the optic nerve (ON) exerts mechanical drag on the eye as it moves through the retrobulbar tissues, so that loading emanates into surrounding tissues from the ON attachment at the optic disc.^4^ Although retrobulbar tissues and orbital tissue pressure may also contribute, previous studies suggest that ON traction is an important contributor to posterior globe deformation during large gaze angles.^4^^–^^6^ Repetitive loading of the disc and nearby tissues might cumulatively remodel or damage them, leading to pathologies including optic neuropathies.^7^^,^^8^

It is well established that large horizontal ductions deform the optic disc and peripapillary sclera and compress the choroid.^9^ Using optical coherence tomography (OCT), Suh et al.^6^ quantified gaze-induced strains and demonstrated substantial deformation within the optic disc. Finite element modeling (FEM) further supports these observations. Using FEM, Wang et al.^10^ predicted that horizontal eye rotations can generate local stresses exceeding those produced by intraocular pressure (IOP) changes at duction angles greater than 13°. Modeling by Jafari et al.^11^^,^^12^ has suggested that ON tethering during adduction becomes particularly pronounced beyond approximately 26°, further amplifying mechanical loading on the posterior globe. Le et al.^13^ subsequently showed that strain magnitude during large-angle adduction decreases with age, consistent with age-related tissue stiffening. Advances in imaging and computational analysis now allow subtle retinal vessel motion and tissue deformation to be quantified reliably.^6^^,^^13^^,^^14^ Using OCT, Wang and colleagues^10^ demonstrated substantial deformation of the optic disc following horizontal gaze shifts. Building on this work, Liu et al.^15^ reported that gaze-evoked deformations are amplified in highly myopic eyes, where strain increases with axial length. Similar increases in gaze-induced strain have also been reported in several disease states, including glaucoma as described by Chuangsuwanich et al.,^8^ thyroid eye disease reported by Fisher et al.,^16^ and optic disc drusen, where Sibony et al.^7^ proposed that repetitive shearing during eye rotations may contribute to disease pathogenesis. More recently, Liu et al.^17^ used computational simulations to demonstrate that globe and ON morphology significantly influence both the magnitude and spatial distribution of gaze-induced optic disc strains. Collectively, these studies reinforce the concept that horizontal eye rotations impose important mechanical loading on the posterior pole.

Conspicuously absent from investigation to date has been the effect of vertical eye rotations. Vertical eye rotations are ubiquitous in our daily lives, occurring constantly during activities such as walking, running, and driving, where clear vision must be maintained despite head and body rotations and translations. Gaze stability under these dynamic conditions is primarily mediated by the VOR, which compensates for vertical as well as horizontal angular and linear head acceleration to preserve retinal image stability during self-motion.^18^^–^^20^ Recent imaging evidence demonstrated that vertical and horizontal eye rotations also alter the configuration of the intraorbital subarachnoid space surrounding the ON.^21^ Age related stiffening of connective tissues may further modify this mechanical environment.^22^^,^^23^ Vertical gaze changes would be expected to impose complex mechanical loads on the posterior globe.^24^^,^^25^ Therefore, understanding the effects of these vertical eye rotations on the optic disc and peripapillary tissues is important, because, if recurrent gaze-induced loading due to horizontal eye rotations is suspected to contribute to ocular pathology, then this suspicion should also extend to vertical eye rotations. Perhaps the cumulative effects of vertical eye rotations also contribute to changes in the structural integrity and remodeling of the disc and peripapillary tissues over time.

The slow acquisition rate of OCT limits its application to repetitive capture in multiple eye positions.^26^ In contrast, confocal scanning laser ophthalmoscopy (cSLO) provides rapids surface imaging suitable for quantifying in-plane motion at high temporal resolution.^27^ When paired with deep-learning segmentation techniques and optical flow algorithms, cSLO enables precise tracking of retinal vessels displacements without manual input.^28^^,^^29^ Park et al.^27^ used cSLO to demonstrate optic disc and peripapillary strain caused by horizontal ductions, but the effects of vertical eye rotations have not been studied to date. The present study aimed to further automate the method and to extend the cSLO approach to ocular tissue strains by including vertical gazes.

## Methods

### Subjects

Healthy volunteers with lightly pigmented eyes were recruited and gave written informed consent under a protocol approved by the Institutional Review Board of the University of California, Los Angeles, and in conformity with the tenets of the Declaration of Helsinki. Lightly pigmented eyes were specifically selected to enable visualization of choroidal vessels. Subjects were excluded if they had limited ocular movements, strabismus due to cranial nerve palsy or restrictive myopathy, high myopia, strabismus surgery within the preceding year, or systemic conditions affecting eye movements. Subjects with comitant small-angle strabismus not limiting ocular ductions or who had undergone strabismus surgery more than 1 year prior were eligible for inclusion.

The main study group consisted of 39 adults, including 10 younger subjects between the ages of 18 and 40 years (mean ± SD, 30 ± 6 years) and 29 older subjects over 40 years (mean ± SD, 67 ± 10 years). The younger group included seven females and three males, and the older group included 18 females and 11 males. Repeatability analysis was performed on a separate group of nine participants with mean age 38 ± 23 years, including one child who was 14 years old, five younger adults, and three older adults

### Imaging

Images were captured in the cSLO mode of the SPECTRALIS camera (Heidelberg Engineering, Heidelberg, Germany) mounted on its incorporated horizontal and vertical gimbals. These images were acquired at 4.7 frames per second, in a mode that is distinct from en face OCT images, which can alternatively be obtained from the same instrument but reconstructed from stacks of two-dimensional (2D) tomographic OCT sections obtained by much longer acquisitions times of up to tens of seconds. For cSLO, the head of each subject was firmly stabilized on the usual chin and forehead support of the scanner, augmented with custom, three-dimensional (3D)-printed adjustable temple pads supported by the chinrest frame to prevent changes in head position in eccentric gazes that would otherwise have compromised the accuracy of the imposed ocular duction angles. For imaging, subjects fixated the internal target of the scanner that was placed to allow imaging of both the disc and the fovea. The scanner was set to high-resolution infrared mode at 100% power with manual focus. The imager was rotated to alter its azimuth or elevation angle to follow the same region of the posterior segment in multiple gaze directions. Scanner azimuth accounted for the 12° nasal offset of its internal target to establish zero-reference yaw position for that eye. This offset was incorporated into a custom goniometer scale affixed to the yaw gimbal of the scanner so the zero-reference position corresponded to straight-ahead gaze. Because the optic disc lies nasal to the visual axis of each eye, this offset necessitated separate angular reference scales for the right and left eyes.

After central gaze imaging, the imager was rotated in yaw by 30° to capture images in adduction and abduction, taking into consideration the horizontal offset of the internal fixation target of the scanner.^6^ For vertical gazes, azimuth was set to the central position for the scanned eye, and the imager was pitched upward or downward by 10°, corresponding to the mechanical limit of the gimbal. Imaging was in the following sequence: central gaze, abduction, adduction, supraduction, and infraduction. Images were exported from the scanner at high resolution (1536 × 1536 pixels) in tagged image file format (TIFF) for analysis with each image covering an area of 8.8 × 8.8 mm^2^ of the posterior eye. Axial length (AL) for each eye was measured using the IOLMaster 500 (Carl Zeiss Meditec, Jena, Germany).

### Image Processing

Uneven illumination across the cSLO imaging field was normalized using heavy Gaussian filtering subtracted in the logarithmic space from the original image to make image brightness appear uniform. Images were segmented automatically into three types of binary masks to select features for analysis: retinal vessels, optic disc, and fovea. Semantic segmentation of these structures was achieved using DeeplabV3+, a supervised deep-learning architecture based on convolutional neural networks (CNNs).^28^^,^^30^ Ground truth annotations were created for CNN training by three experienced ophthalmologists who manually traced each feature in 104 illumination-corrected images (1536 × 1536 pixels). This high resolution of these images enabled precise labeling of fine vascular structures.

**Figure 1.**
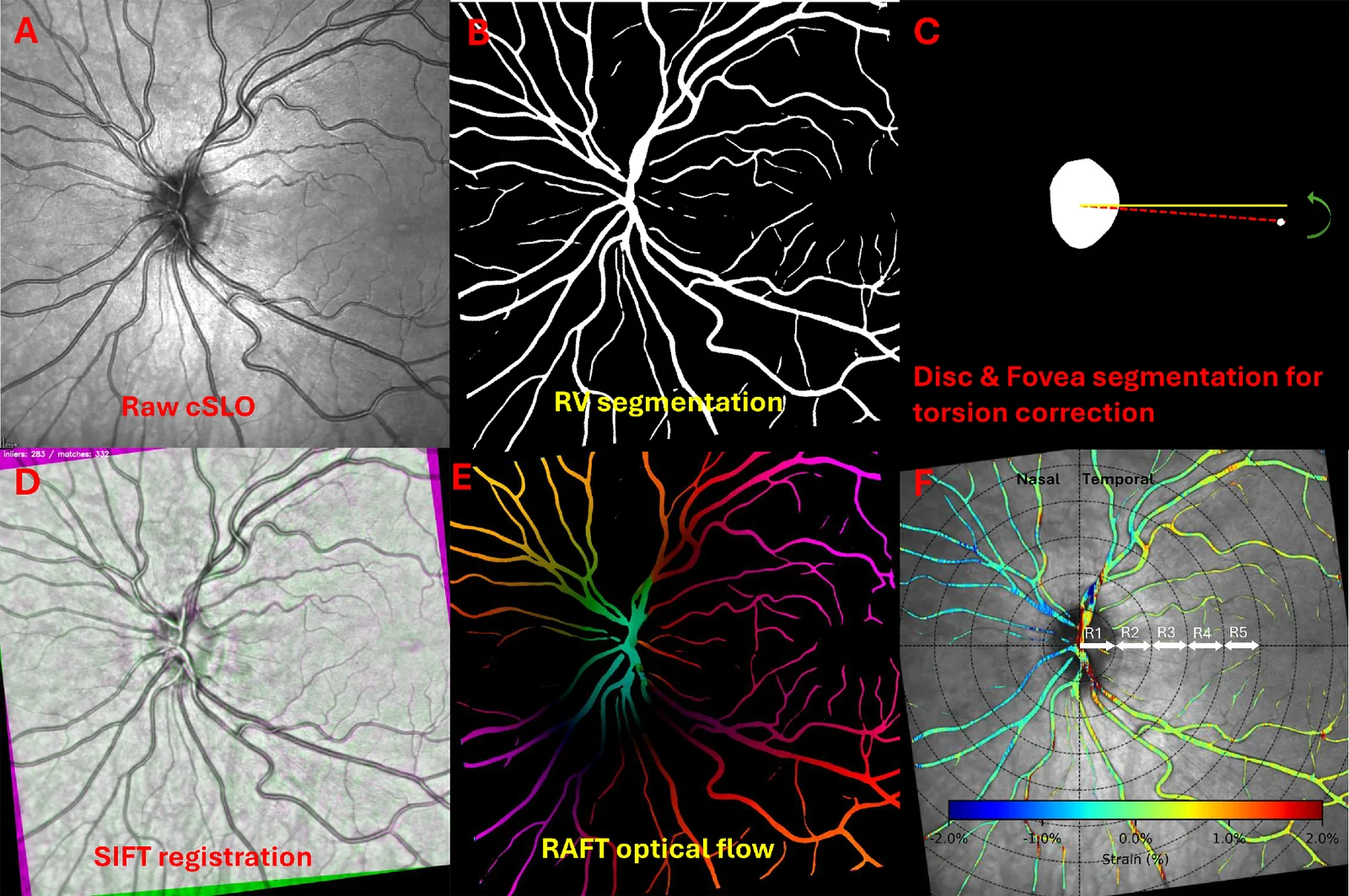
Imaging processing pipeline. (**A**) Original cSLO image. (**B**) Retinal vessel segmentation using deep learning. (**C**) Optic disc and fovea segmentation using deep learning for torsion correction. (**D**) SIFT registration to align eccentric gazes against primary gaze. (**E**) RAFT optical flow to compute displacements in eccentric gaze. (**F**) Strain heat map.

The 104-image training set was augmented to 5000 by flipping, rotation, contrast and brightness adjustments, and noise addition. Training was conducted for 25 epochs using a weighted BCEwithLogitLoss function with weights of 30, 20, and 90 assigned to the retinal vessel, optic disc, and fovea segmentation models, respectively (Fig. 1). Three separate models were trained, each specialized for one of the foregoing features of interest. Segmentation performance was assessed using the Dice coefficient complemented by area under the receiver operating characteristic curve (AUROC). AUROC evaluated the ability of a model to distinguish features from the background across varying decision thresholds, whereas the Dice coefficient quantifies spatial overlap between the predicted and reference segmentations, reflecting balance between precision and recall. Despite applying illumination correction, the visibility of fine vessels remained strongly dependent on the image exposure. These smaller vessels contribute limited spatial information and are more vulnerable to noise and motion artifacts, which reduce their reliability for strain analysis. Consequently, segmentation efforts were focused on well-defined, high-contrast retinal vessels. This constraint reduces sensitivity to fine vasculature but lowers false-positive rates and improves the robustness of downstream optical flow analysis. For fovea segmentation, the training set included images with both clearly delineated and poorly visible or absent foveal structures to enhance model generalizability.

### Torsion Correction

To anatomically standardize spatial orientation,^31^ each image was rotated by the disc–foveal angle so that the fovea was horizontally aligned with the optic disc center. The disc–foveal angle was calculated from centroid of the optic disc and fovea masks. When automatic foveal segmentation failed due to indistinct or absent foveal features, manual input was used to define the fovea center. The centroid of each mask was determined to calculate the angle formed by the disc–foveal line with the horizontal. Each image was then rotated about the optic disc center to correct ocular torsion. This automated alignment minimized human-induced bias (Fig. 1).

For standardization, all images were analyzed as if in left eye orientation by horizontal reflection of right-eye images. The entire analysis pipeline was fully automated, accepting images as input and generating output without manual intervention, except in rare cases where an anatomically indistinct fovea necessitated manual fovea selection because of automated segmentation failure.

### Registration

To compensate for large rigid-body motion arising from variation in scanner alignment, torsion-corrected eccentric gaze images were registered to the corresponding central gaze image. A rigid affine transformation matrix was obtained using the scale-invariant feature transform (SIFT) algorithm,^32^ which detects distinctive keypoints and descriptors for robust alignment despite varying noise and illumination. Only rotation and translation were registered, without warping or shearing, to ensure that residual displacements represented true deformation induced by gaze change (Fig. 1).

### Optical Flow Implementation

Residual displacements of retinal vessels were quantified using Recurrent All-Pairs Field Transforms (RAFT), a deep-learning optical flow algorithm for dense motion estimation. A pretrained RAFT model developed by Teed and Deng^29^ was used without additional fine-tuning, given its sensitivity to small and rapid displacements. Each registered eccentric gaze image was compared to the central gaze image, and pixelwise changes were computed to generate dense displacement field maps. Retinal vessel masks were then applied to isolate pixels associated with vessel motion and to exclude other motion (Fig. 1).

### Strain Computation

Retinal vessel displacements were converted to strain fields using principles of continuum mechanics. The central gaze image was treated as the undeformed reference state, and each eccentric gaze image was considered a deformed state. Displacement vectors derived from optical flow indicated deformation between these two states and allowed computation of the Green–Lagrange strain tensor by evaluating the spatial gradients of displacement, yielding horizontal (*E_xx_*), vertical (*E_yy_*), and shear (*E_xy_*) strain components.^33^ The von Mises equivalent strain (*E_eq_*) was calculated under plane strain condition assuming Poisson's ratio 0.5 according to Equation 1.

(1) $$\begin{array}{c} E_{eq}=\frac{2}{3}\sqrt{\left(\frac{3}{2}\left(E_{xx}^{2}+E_{yy}^{2}\right)+3E_{xy}^{2}\right)}\; \end{array}$$

Equivalent strain is a scalar quantity representing the magnitude of shape distortion independent of orientation, obtained by removing the volumetric component of strain. It helps detect relative changes or patterns of deformation rather than fully characterize material properties.

Any apparent strain value exceeding 25% was discarded from the overall analysis, as such a high strain is physiologically impossible and would represent only noise. The influence of the cardiac pulse on mean gaze-induced strain was negligible relative to the measured deformations.^34^ Because retinal vessels are embedded within the retinal tissue, their displacement was assumed to approximate local retinal deformation. The term “strain” here refers to apparent strain in the image plane calculated from projected vessel displacements measured in en face cSLO images. This quantity represents projected in-plane deformation and is not the complete 3D tissue strain.

### Strain Representation

Computed strains were represented in a coordinate system centered at the optic disc. The radius of a nominally circular optic disc was estimated from the area of the disc mask and then used to partition each image into four concentric annular regions of width equal to one disc radius. Each region was then bisected by a vertical line through the disc centroid to demarcate nasal and temporal hemisectors for analysis. For qualitative visualization, strain values were overlaid on the corresponding central gaze images to generate heatmaps depicting both magnitude and direction of deformation (Fig. 1). In addition to vessel-specific strain heatmaps, full-field heatmaps were generated to evaluate the presence of motion artifacts such as saccades or image jitters. After the strain analysis, physiological vessel deformation produced consistent and spatially coherent strain patterns. In contrast, motion artifacts resulted in systematic horizontal bandlike structures across the entire image inconsistent with vessel deformation. Gaze frames exhibiting such artifacts were excluded from analysis or reacquired when possible.

### Repeatability

To evaluate the repeatability of strain analysis, the complete image processing pipeline was repeated for nine subjects who were not members of the original two groups but underwent the imaging protocol in duplicate. For each participant in the reproducibility study, cSLO images at all eccentric gaze positions were acquired twice, with a 30-minute interval between acquisition sets. Rather than using central gaze as a common reference, the initially acquired image in each gaze position was compared against the second acquisition in that same gaze position. In this situation, ideal strain should be zero for repeated imaging in the same gazes. Realistically, any nonzero computed strain would be attributable to noise in the method. Subsequent analysis was identical to the main analysis. Mean repeatability values were averaged across subjects for each region analyzed. The greater of the absolute maximum strains in either the nasal or temporal hemisectors was conservatively defined as the repeatability error band for the entire region. This approach was applied to all calculated strains.

### Validation on Prior Dataset

The current automated analysis pipeline was validated by re-analysis of the dataset from our published study for horizontal gazes.^27^ In the earlier method, optic disc center, disc radius, and fovea center had been manually selected. The repeatability of these steps in a manual process was not validated in the prior study, but the current automated re-analysis indicated that these manually measured parameters influence topographic assignment of strain values to specific regions and hemisectors. For comparison with the current automatic workflow, the previously published dataset was reanalyzed using the current method differing only in that manually input coordinates for the optic disc and fovea were replaced with automatically derived values.

### Strain Definitions

Tensile strains were taken as positive and compressive strains as negative, following standard sign conventions. For shear strain, positive values corresponded to an increase in vertical displacement (positive downward) moving from left to right, whereas negative values indicated decreased vertical displacement moving from left to right. Because equivalent strain reflects overall deformation magnitude independent of direction, it served as one of the primary metrics for evaluating gaze-induced mechanical loading. Individual strain components (horizontal, *E_xx_*; vertical, *E_yy_*; and shear, *E_xy_*) were also analyzed for the directionality of loading.

### Statistical Analysis

Generalized estimating equations were used in SPSS Statistics (IBM Corporation, Chicago, IL, USA) to evaluate the effects of gaze direction, age group, axial length, and retinal region on each strain component. This approach accounts for interocular correlation between eyes of individual subjects and has type 1 error characteristics superior to *t*-testing.^35^ Statistical significance was taken at 0.05. Graphical representations were generated using Prism 10 (GraphPad Software, Boston, MA, USA).

## Results

### Repeatability

The repeatability errors across eccentric gazes for all strain components are summarized in Table 1. Horizontal strain (*E_xx_*) exhibited error bounds ranging from ±0.01% to ±0.11% in adduction, ±0.17% to ±0.23% in abduction, ±0.04% to ±0.13% in infraduction, and ±0.05% to ±0.11% in supraduction. Vertical strain (*E_yy_*) exhibited similar variability, with error ranges of ±0.06% to ±0.18% in adduction, ±0.05% to ±0.31% in abduction, ±0.03% to ±0.18% in infraduction, and ±0.04% to ±0.20% in supraduction. The repeatability error for shear strain (*E_xy_*) was between ±0.03% and ±0.15% in adduction, ±0.04% in abduction, ±0.05% in infraduction, and ±0.05% to ±0.13% in supraduction. Equivalent strain (*E_eq_*), reflecting overall deformation magnitude, exhibited repeatability errors ranging from ±0.48% to ±0.80% in adduction, ±0.50% to ±0.99% in abduction, ±0.43% to ±0.58% in infraduction, and ±0.46% to ±0.91% in supraduction. Despite these repeatability error bounds, measured strain magnitudes within the optic disc consistently exceeded the error range, supporting that observed deformations represent true physiological signals rather than measurement noise.

**Table 1.** Repeatability

	Error Range (%)			
Strain Component	Adduction	Abduction	Infraduction	Supraduction
ε_*xx*_	±0.01 to ±0.11	±0.17 to ±0.23	±0.04 to ±0.13	±0.05 to ±0.11
ε_*yy*_	±0.06 to ±0.18	±0.05 to ±0.31	±0.03 to ±0.18	±0.04 to ±0.20
ε_*xy*_	±0.03 to ±0.15	±0.04	±0.05	±0.05 to ±0.13
ε_*eq*_	±0.48 to ±0.80	±0.50 to ±0.99	±0.43 to ±0.58	±0.46 to ±0.91

### Segmentation Performance

The model segmentation performance metrics for each feature are summarized in Table 2.

**Table 2.** Model Segmentation Performance Metrics

Feature	Test AUROC	F1/Dice Coefficient Score
Retinal vessels	0.97	0.82
Optic disc	0.99	0.96
Fovea	0.97	0.88

### Equivalent Strain During Vertical Ductions

**Figure 2.**
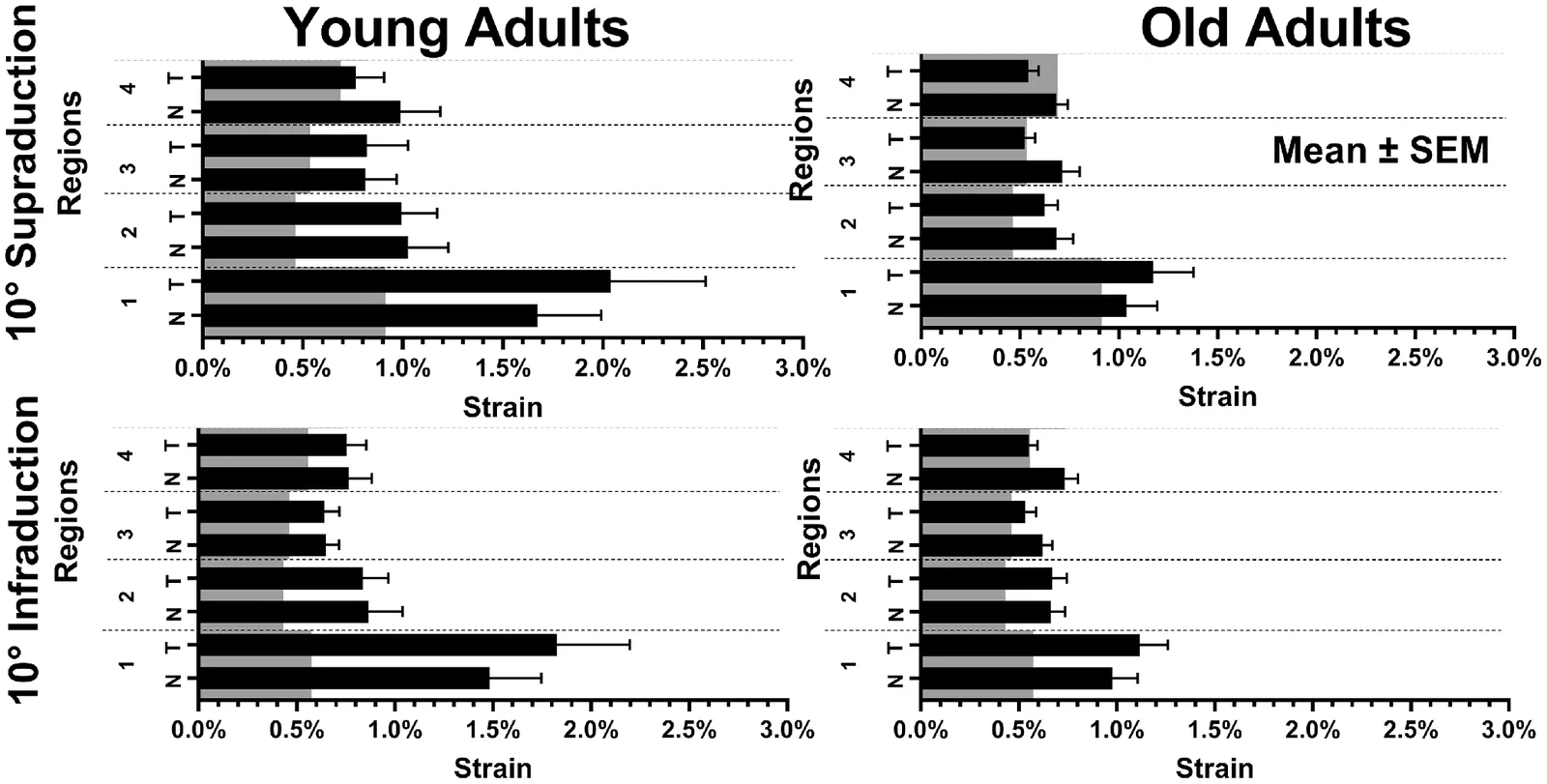
Equivalent strain during supraduction and infraduction. N, nasal; T, temporal.

Vertical ductions strained retinal vessels and peripapillary tissues. Equivalent strain in retinal vessels during supraduction and infraduction was predominantly concentrated within the optic disc in both younger and older subjects. Strains decreased sharply from the disc (region 1) to the adjacent region 2 (*P* < 0.001), then declined progressively with increasing distance from the disc. In young adults, supraduction produced the highest equivalent strain within the disc (region 1), reaching 2.03% ± 0.47% (SEM) in the temporal and 1.67% ± 0.31% in the nasal hemisector. Equivalent strains were similar during infraduction, with peak strains of 1.82% ± 0.37% in the temporal and 1.43% ± 0.26% in the nasal hemisector. In older adults, equivalent strains within the optic disc were significantly lower than those in younger adults (*P* = 0.03). During supraduction, strains in the disc reached 1.17% ± 0.20% on the temporal side and 1.03% ± 0.15% on the nasal side, and infraduction resulted in peak values at 1.11% ± 0.14% on the temporal side and 0.97% ± 0.15% on the nasal side (Fig. 2). In both age groups and for both supraduction and infraduction, strains within the temporal optic disc were consistently higher than in the nasal disc (*P* = 0.04). Although equivalent strains during supraduction and infraduction did not differ significantly from one another within the optic disc, both significantly exceeded zero for both young and older adults (*P* < 0.001).

### Horizontal Strain During Vertical Duction

**Figure 3.**
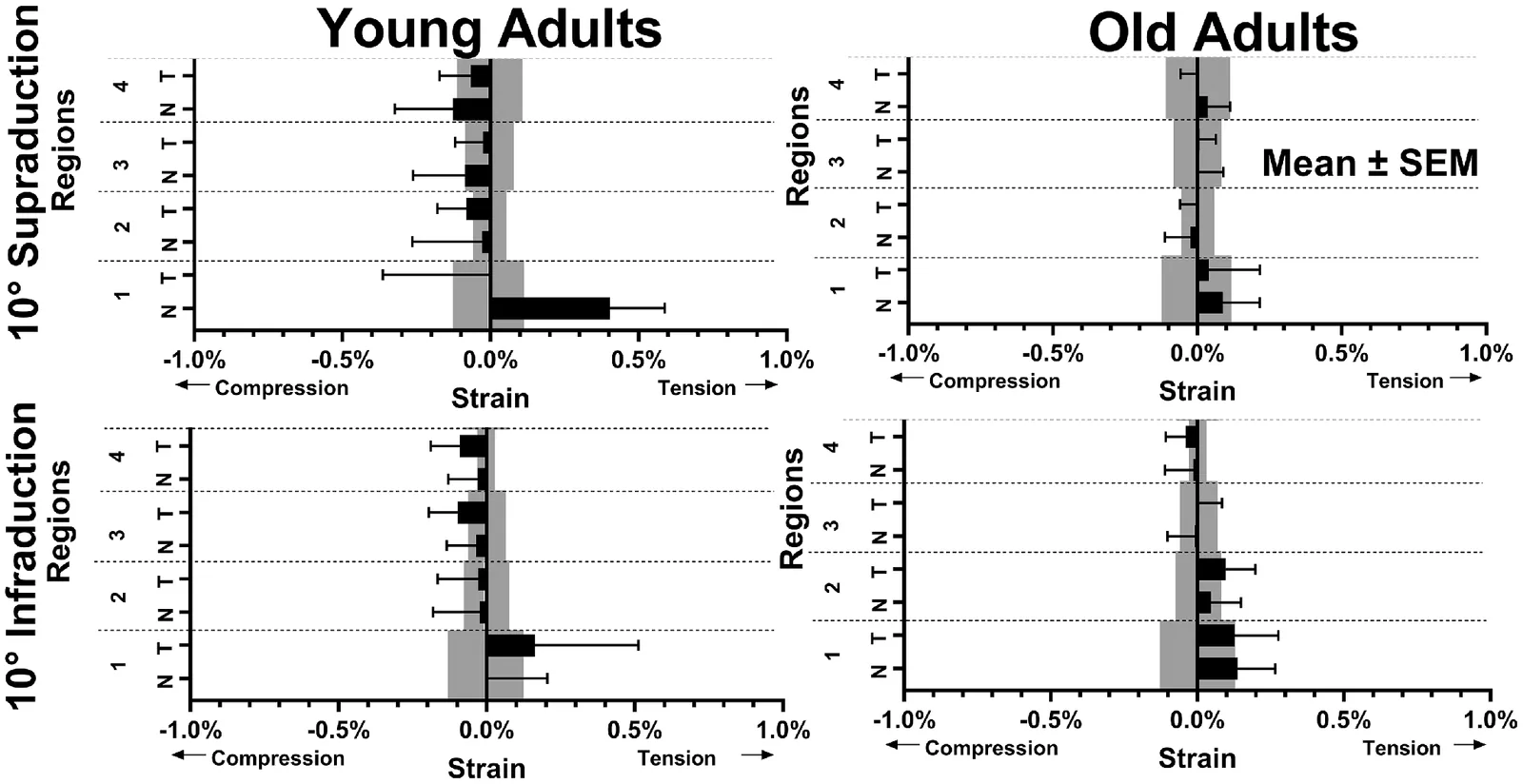
Horizontal strain during supraduction and infraduction.

Horizontal strain during vertical duction was below the noise threshold in all regions for both younger and older adults during supraduction and infraduction (Fig. 3).

### Vertical Strain During Vertical Duction

**Figure 4.**
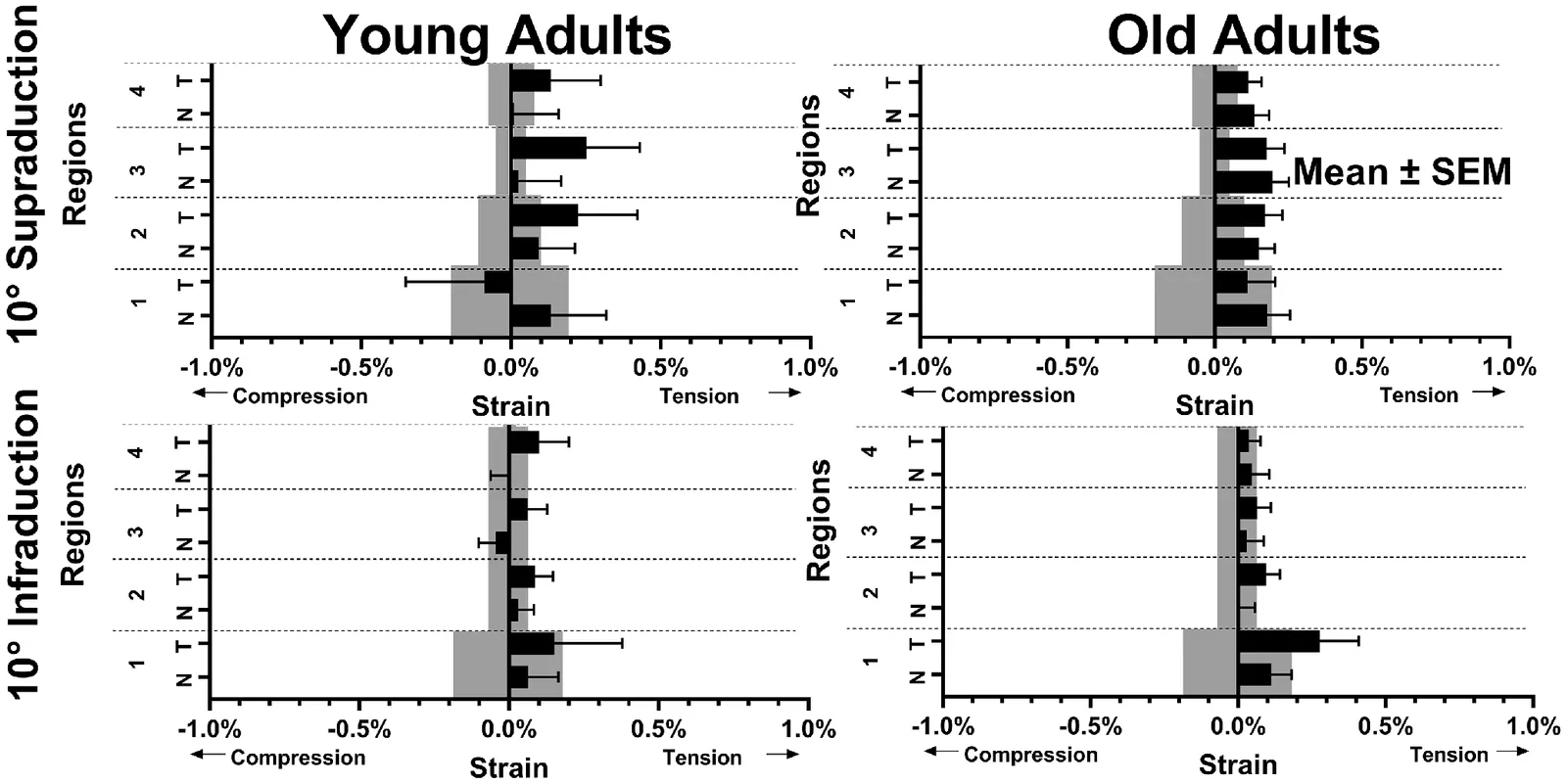
Vertical strain during supraduction and infraduction.

Vertical duction produced measurable vertical strain, which was greatest during infraduction on the temporal side of the optic disc in older adults (0.27% ± 0.13%) (Fig. 4). However, during infraduction, vertical strain in other regions was not significant in either age group. In contrast, during supraduction, vertical strain within the optic disc did not exceed the repeatability threshold but was significant in both groups in region 2 and beyond. Nearly all significant vertical strains during supraduction were tensile.

### Shear Strain During Vertical Gazes

**Figure 5.**
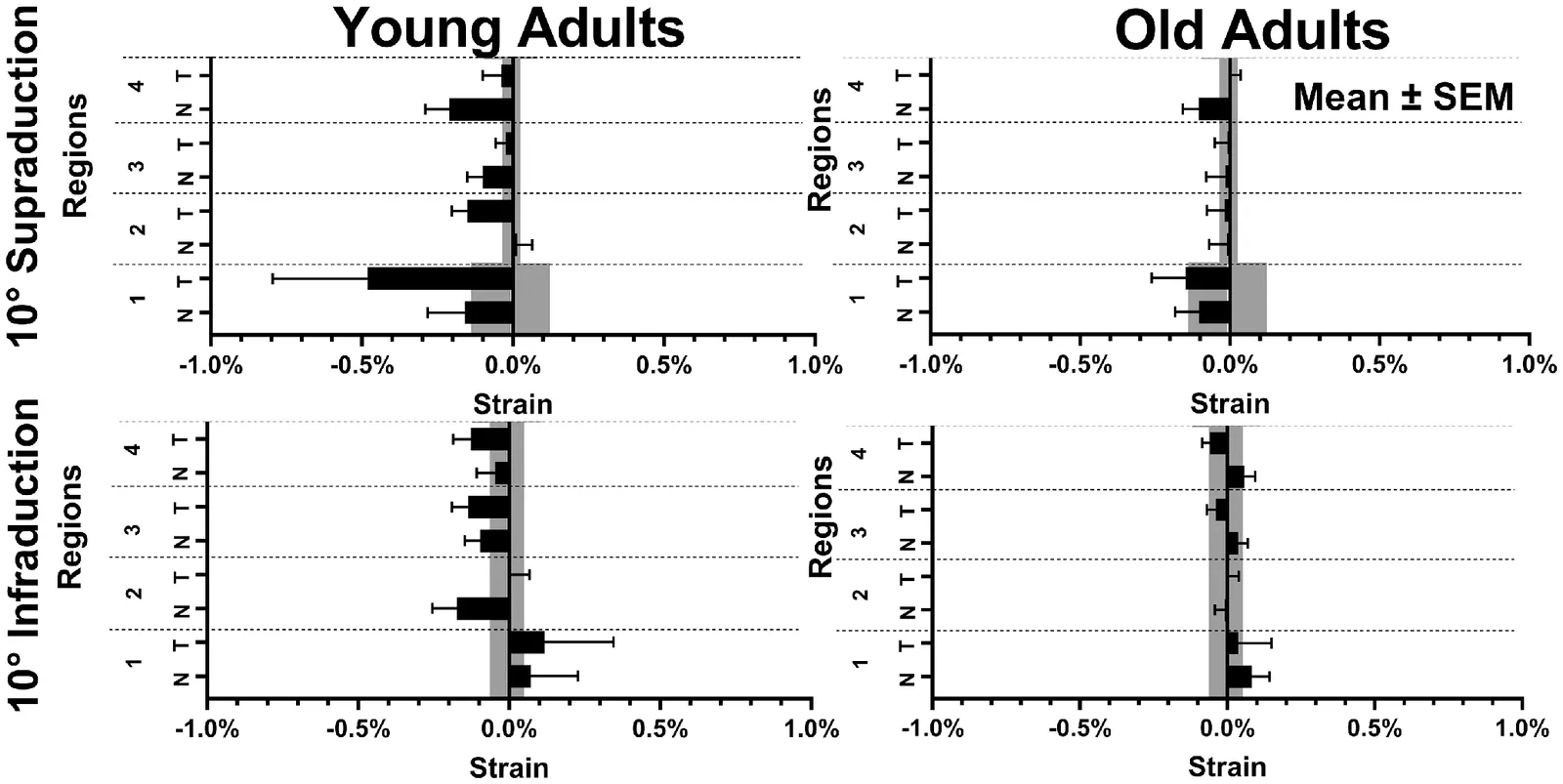
Shear strain during supraduction and infraduction.

Strain during vertical gazes was predominantly due to shear. In young adults, supraduction produced pronounced −0.48% ± 0.31% shear strain on the temporal side of the optic disc that was significantly greater than during infraduction at 0.11% ± 0.22% (*P* = 0.001). In contrast, shear strain in older adults was negligible during both supraduction and infraduction. Shear strain in younger adults significantly exceeded zero in regions 1 and 2, with maximum values reaching −0.48% ± 0.31% during supraduction (*P* = 0.023) and −0.17% ± 0.08% during infraduction (*P* = 0.032) (Fig. 5).

**Figure 6.**
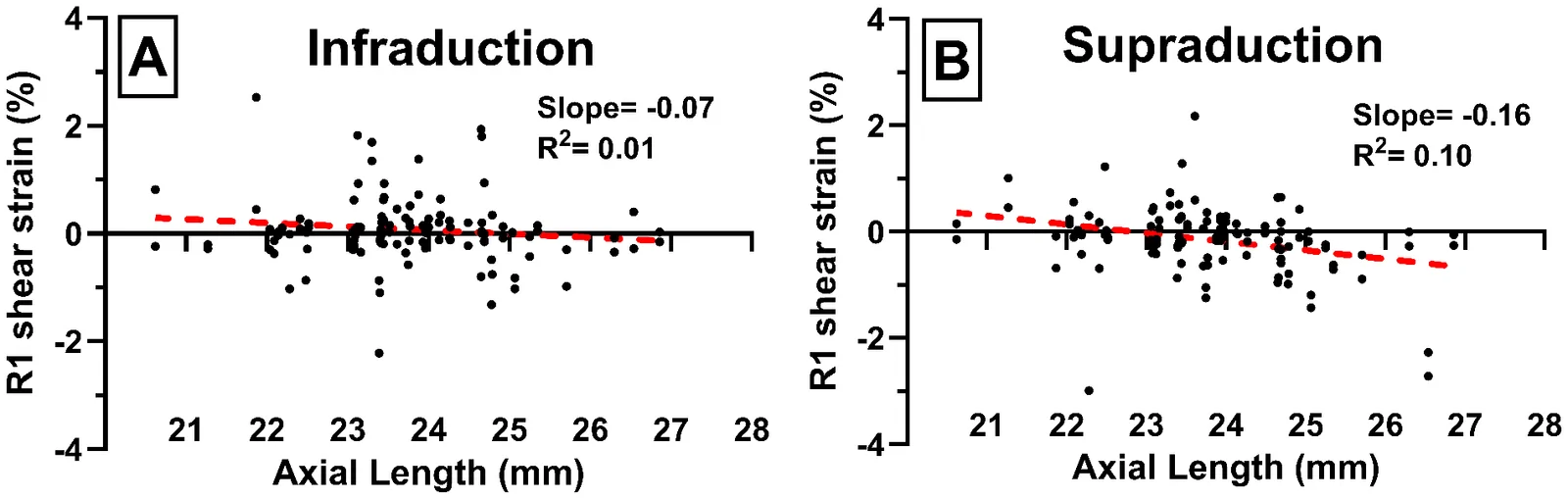
(**A, B**) Shear strain in region 1, the optic disc, as a function of AL during infraduction (**A**) and supraduction (**B**).

Axial length (AL) was weakly but significantly associated with shear strain inside the disc (region 1), which decreased with increasing AL. Linear regression showed that shear strain decreased by approximately 0.07% per millimeter increase in AL during infraduction (*P* = 0.018, *R*^2^ = 0.014) and by approximately 0.16% per millimeter increase during supraduction (*P* = 0.0005, *R*^2^ = 0.10) (Fig. 6). Although statistically significant, the low coefficients of determination indicate that AL explains only a small proportion of the variance in shear strain.

### Equivalent Strain During Horizontal Gazes

**Figure 7.**
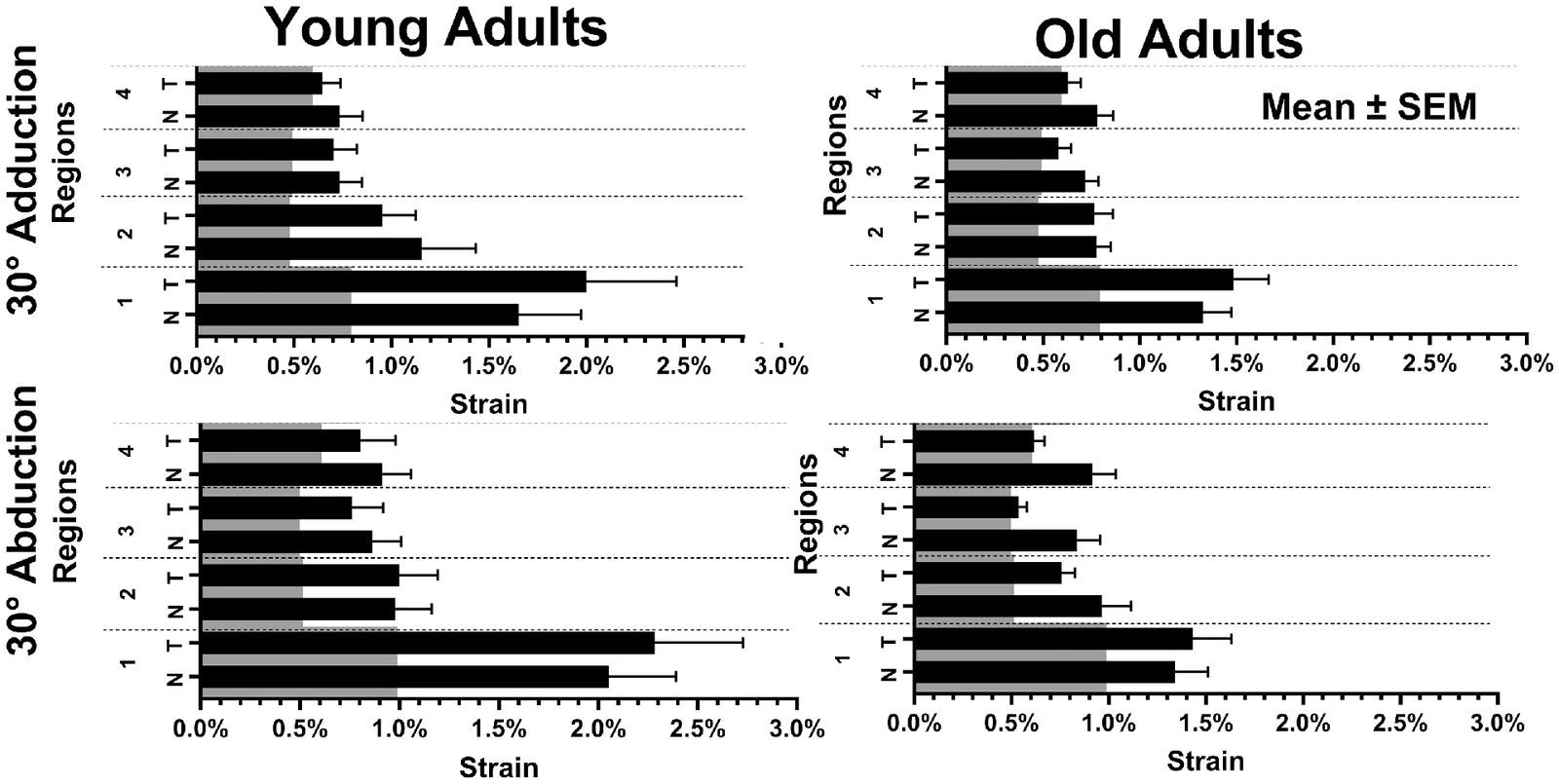
Equivalent strain during abduction and adduction.

Vessels inside the optic disc (region 1) exhibited significantly greater strains than those in peripheral regions in both age groups during abduction and adduction (*P* < 0.001). During adduction, equivalent strain in the temporal side of the disc reached 1.99% ± 0.46% in young adults, similar to 1.48% ± 0.18% in older adults. During abduction, young adults exhibited higher strains within the disc, reaching 2.28% ± 0.44% temporally and 2.05% ± 0.33% nasally, similar to values in older adults of 1.43% ± 0.97% temporally and 1.34% ± 0.17% nasally. For both abduction and adduction, strain within the optic disc was significant but similar in both younger and older subjects (*P* < 0.001 for both gazes) (Fig. 7).

### Horizontal Strain During Horizontal Gazes

**Figure 8.**
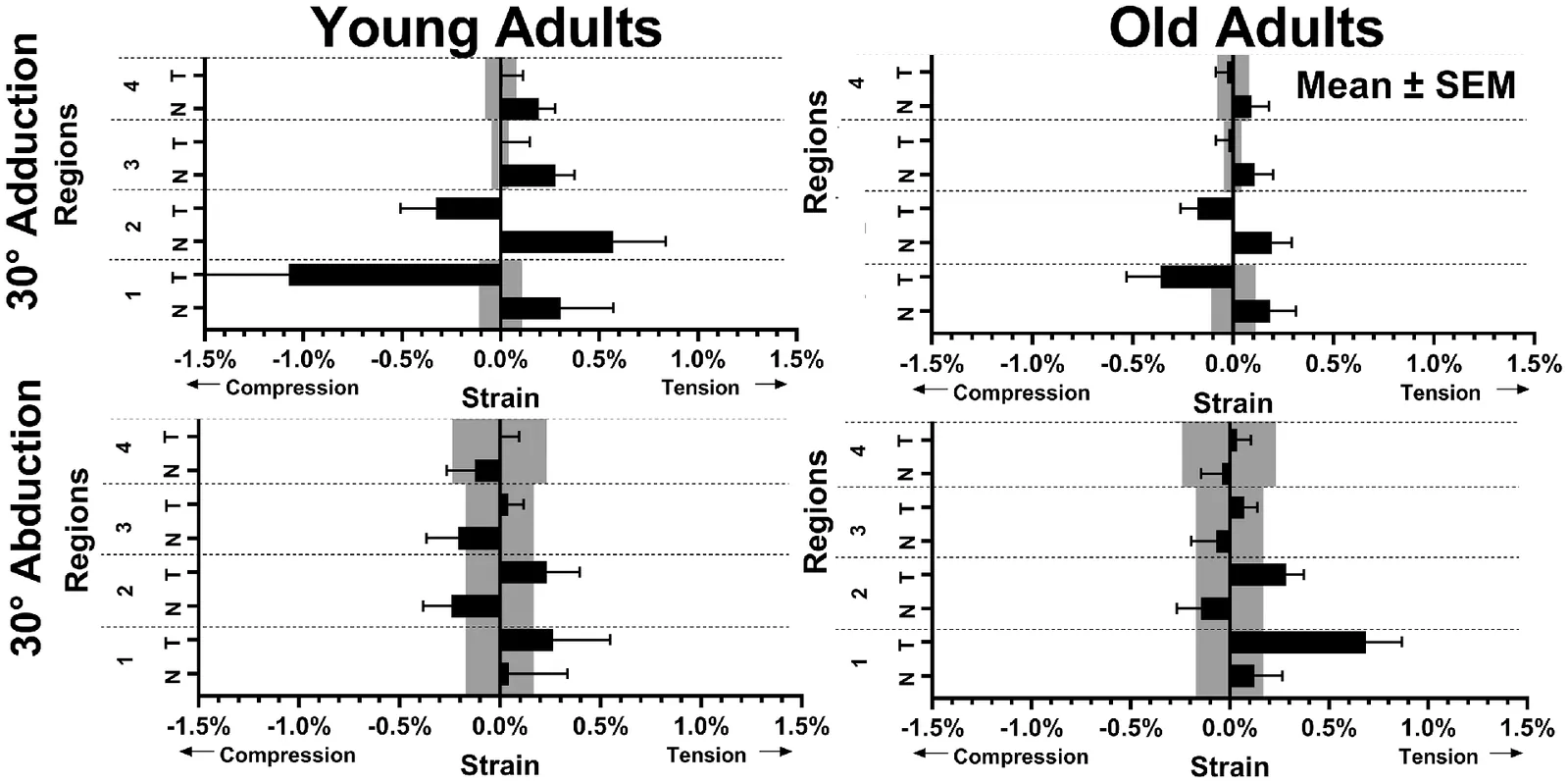
Horizontal strain during abduction and adduction.

Horizontal duction caused horizontal strain within the optic disc. Horizontal vascular strain within the disc was significantly greater on the temporal side during adduction in younger adults (−1.07% ± 0.28%) than older adults (−0.36% ± 0.17%; *P* = 0.005) in the same gaze direction and hemisector. Although abduction generated smaller horizontal strains than adduction, it was significantly nonzero within regions 1 and 2 (*P* = 0.012), with maximum horizontal strain reaching 0.68% ± 0.18% in the temporal optic disc in older adults. In both age groups, horizontal strain decreased progressively with distance from the optic disc, especially in adduction. Notably, in regions 1 and 2, the direction of horizontal strain on the temporal side reversed from compressive in adduction to tensile in abduction for both the age groups (Fig. 8).

**Figure 9.**
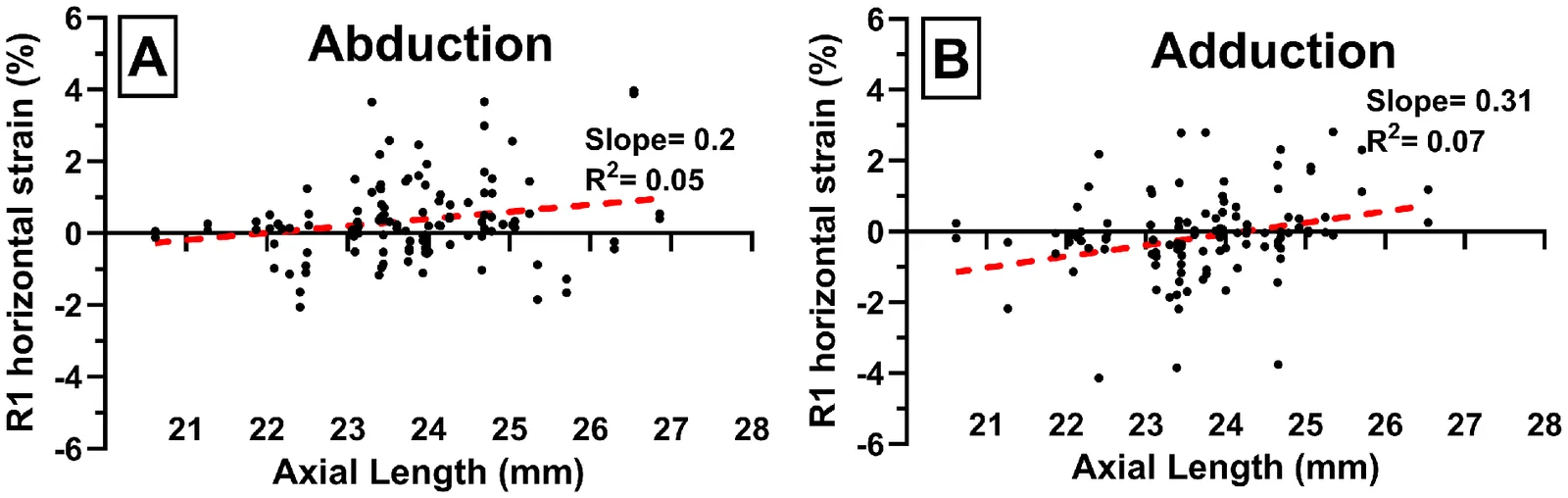
(**A, B**) Horizontal strain in region 1, the optic disc, as a function of AL during abduction (**A**) and adduction (**B**).

AL was significantly but weakly associated with horizontal strain in the optic disc (region 1). Linear regression demonstrated a positive relationship between AL and horizontal strain during abduction (*P* = 0.018, *R*^2^ = 0.046) and adduction (*P* = 0.005, *R*^2^ = 0.07). Horizontal strain increased by approximately 0.2% per millimeter of AL during abduction and 0.31% per millimeter during adduction (Fig. 9).

### Vertical Strain in Horizontal Gazes

Horizontal ductions did not generate significant vertical strain, which generally remained within the repeatability error bands in all regions, except the temporal side of the optic disc (region 1) during abduction in young adults, where vertical strain reached −0.84% ± 0.43% and was significantly greater than in older adults (0.28% ± 0.14%; *P* = 0.003).

### Shear Strain in Horizontal Gazes

There was no significant shear strain in any region during horizontal ductions in any subject group.

## Discussion

This study exploited high-resolution, cSLO imaging with deep learning and digital image processing to accurately measure in-plane retinal vessel displacement from central to multiple secondary gazes, allowing quantification of strains likely to reflect strains in the transparent retina in which the vessels are embedded. Unlike the previously reported manual method of cSLO analysis,^27^ the current approach was completely automated, eliminating user input with potential for bias. Findings generally confirm prior reports that horizontal ductions induce mechanical strains in the disc and peripapillary region^7^^,^^10^^,^^36^ and extend these findings to show that even modest vertical duction produces comparable strains. A striking, novel finding of this study is that the magnitude of equivalent strain in the optic disc and peripapillary retinal vessels is similar for 10° vertical duction to the strain induced by 30° horizontal duction.

Strain due to both horizontal and vertical duction is greatest within the optic disc and decreases monotonically with distance from it. This is consistent with loading of the eye by the ON during eye rotation, particularly during large adduction, where the limited length of the ON may tether the globe.^37^^,^^38^ Strain during vertical duction is lower in older adults than younger adults. Horizontal strain increased with axial ocular length, consistent with the findings of Liu et al.^15^

### Vertical Duction

This study revealed strain patterns for supraduction and infraduction. Equivalent strain is concentrated inside the optic disc, where strain is significantly larger than in the neighboring peripapillary retinal regions, consistent with horizontal gazes. The ON is generally in a sinuous configuration during most eye rotations,^39^ except when it tethers during adduction.^37^ Even in a sinuous configuration, the ON can load the eye at the junction of its entry into the globe just posterior to the optic disc, concentrating stress there. This is evident from the equivalent strain plots for both supraduction and infraduction (Fig. 2), which show that strain routinely reaches about 2%, declining to about 1% in the immediate peripapillary region. The decrease in strain between these regions can be associated with differing tissue properties of the optic disc and posterior sclera. The posterior sclera is significantly stiffer than the ON,^40^^,^^41^ leading to about half the amount of strain developed in it than in the optic disc.^42^

The approximately 2% apparent image-plane equivalent strain observed within the optic disc indicates that vertical eye movements generate substantial mechanical deformation at this site. Experimental studies of acute IOP elevation have reported local tissue strains in the posterior eye that overlap the range observed in the present study,^43^^,^^44^ although the exact values depend on the tissue examined, loading conditions, and strain metric employed. Because the present measurements were derived from 2D images rather than volumetric tissue deformation, the quantities are not exactly comparable. Nevertheless, the similar strain magnitudes suggest that physiological eye movements may represent a biomechanically important loading mechanism alongside IOP. Unlike elevations in IOP that occur under specific physiological or pathological conditions, eye movements occur thousands of times each day, including frequent vertical gaze shifts during normal visual behavior and VOR activity. Consequently, optic disc tissues are likely exposed repeatedly to these mechanical loads throughout daily life, making gaze-induced deformation an important cumulative biomechanical stimulus. It is notable that approximately 2% equivalent strain inside the temporal optic disc in younger adults is similar in both supraduction and infraduction. The observed symmetry in strain may be related to the approximately symmetric vertical entry of the ON into the globe.^45^ This contrasts with large horizontal asymmetry, where the ON enters substantially nasal to the fovea.

Despite similarity of equivalent strain in infraduction and supraduction, individual strain components exhibit varying patterns. High horizontal and shear strains are significant during supraduction but not infraduction (Figs. 3, 5). Unlike adduction, tethering of the ON does not generally occur during even large vertical ductions. It has been proposed that progressive, age-related temporal tilting and torsion of the optic disc may be related to ON loading during eye rotations.^6^^,^^14^ The optic disc is more commonly tilted temporally than vertically,^14^ and, if this is related to remodeling due to accumulated effects of eye rotations, it might be because disc strains during vertical ductions are symmetrical and thus balance or cancel during quotidian sequences of upward and downward eye rotations. However, with aging, supraduction becomes progressively limited, but infraduction does not.^46^

AL influences strain generation within the optic disc and surrounding tissues during vertical and horizontal eye rotations, as influenced by globe size and shape. Longer, highly myopic eyes exhibit greater horizontal optic disc strains during adduction and abduction, consistent with the findings reported by Liu et al.^15^ In contrast, shorter eyes tend to concentrate strain differently with higher shear strain during supraduction and infraduction due to differences in globe geometry and tissue compliance, suggesting that smaller globes may be more susceptible to shear loading in vertical gazes. However, these effects are minor for the relatively small vertical ductions studied here (Fig. 6).

### Effect of Age

The effect of age is most evident in equivalent strain plots during duction, particularly within the optic disc, where strain magnitudes were generally lower in older than younger adults in all secondary gazes (Figs. 2, 7). In some cases, strain in older adults was about half that in younger adults (Fig. 2, region 1 during vertical duction), probably reflecting tissue stiffening with age.^13^ Human peripapillary sclera is structurally anisotropic^47^^–^^52^ due to collagen fiber orientation.

As the result of age-related tissue stiffening,^53^^–^^59^ strain under the same loading is less, as most of the forces generated by eye rotations are converted to internal tissue stress. In contrast, in younger individuals, the tissues are more compliant and flexible, allowing these forces to propagate more distantly as elastic deformations that represent strain with lower internal stress. Accumulated stress due to eye rotations may contribute to permanent structural changes within the optic disc and peripapillary regions,^7^ where deformation magnitudes are greatest. The overall pattern and direction of strain developed are consistent between younger and older people, suggesting that the force driving deformation is independent of age and the principal effect of age is on strain magnitude in response to this force.

### Re-Examination of Horizontal Duction

Consistent with prior literature,^4^^,^^10^^,^^13^^,^^36^^,^^37^^,^^60^ the present study confirmed that the greatest strain during horizontal duction develops within the optic disc and decreases progressively distance from it. In the current study (Fig. 8), horizontal strain for abduction and adduction was higher on the temporal side of the optic disc than on the nasal side, unlike the report by Park et al.^27^ Our contrary findings in this regard are consistent with theoretical studies.^5^^,^^36^^,^^38^^,^^60^^,^^61^ A possible explanation for this discrepancy may relate to differences in how the fovea and optic disc centers were determined, as these entities define the coordinate system used to divide strain into nasal and temporal regions. Localization differences in the optic disc center shift the origin of the radial regions, and, because the fovea is used to determine the image orientation, even slight differences in the foveal localization introduce small rotational offsets. Because the largest strains in our data were concentrated within the optic disc itself, small angular or translational shifts in the coordinate system can substantially alter how these strains are allocated into nasal versus temporal regions. Unlike the manual boundary delineation used by Park et al.,^27^ the present study employed machine learning based segmentation to automatically identify the fovea and optic disc. Re-analysis of the data from Park et al.^27^ suggests that even small offsets in the optic disc center localization can significantly alter the assignment of strain along the nasal–temporal axis. Differences in subject populations may also contribute to the observed discrepancy. Variations in age, refractive status, and ON head morphology probably also influence the biomechanical response of the posterior eye to gaze-induced loading.
